# A Temperature Sensor Based on a Polymer Optical Fiber Macro-Bend

**DOI:** 10.3390/s131013076

**Published:** 2013-09-26

**Authors:** Alberto Tapetado Moraleda, Carmen Vázquez García, Joseba Zubia Zaballa, Jon Arrue

**Affiliations:** 1 Departamento de Tecnología Electrónica, Universidad Carlos III de Madrid, Avenida de la Universidad 30, Leganés 28911, Spain; E-Mail: cvazquez@ing.uc3m.es; 2 Departamento de Electrónica y Telecomunicaciones, Universidad del País Vasco, Euskal-Herriko Unibertsitatea, Alameda de Urquijo S/N, Bilbao 48013, Spain; E-Mails: joseba.zubia@ehu.es (J.Z.Z.); jon.arrue@ehu.es (J.A.)

**Keywords:** polymer optical fiber sensor, temperature, intensity, bend loss, metal surface

## Abstract

The design and development of a plastic optical fiber (POF) macrobend temperature sensor is presented. The sensor has a linear response *versus* temperature at a fixed bend radius, with a sensitivity of 1.92·10^−3^ (°C)^−1^. The sensor system used a dummy fiber-optic sensor for reference purposes having a resolution below 0.3 °C. A comprehensive experimental analysis was carried out to provide insight into the effect of different surrounding media on practical macro-bend POF sensor implementation. Experimental results are successfully compared with bend loss calculations.

## 1. Introduction

The field of temperature sensors covers a high percentage of today's World sensors market due to the large number of application in which it is necessary to measure temperature, for instance: the automotive industry, air-conditioning control and the chemical industry, among others. Nevertheless, traditional temperature sensors such as thermocouples and thermistors are not well suited for use whenever specific needs are required. This is the case of temperature measurement in many low-cost industrial processes in the presence of electromagnetic disturbances. To solve these problems, innovative solutions based on amplitude or phase fiber-optic techniques have been developed [1]. Temperature phase sensors are based on phase changes of an electromagnetic wave traveling along an optical fiber caused by temperature changes. Different interferometric configurations have been proposed as temperature sensor such as Mach-Zehnder [2] and Fabry-Perot one [3]. Although they usually have larger sensitivities, they require more complex reception circuits. Temperature amplitude modulation sensors are based on optical power variations. Some proposed sensors are based on light attenuation [4], frustration of total internal reflection [5–8], controlled mode coupling [9], light generation [10], Fiber Bragg Grating wavelength shifts [11,12], and fluorescence [13,14]. These sensors have different degrees of complexity that depend on their configuration. All intensity sensors need a reference technique [9,15–17] to avoid false readings caused by fluctuations of the light source, optical fiber attenuation coefficient or other undesired losses.

In this paper, the authors propose and characterize a low-cost intensity macro-bend temperature sensor based on polymer optical fiber (POF). The sensor system used a dummy fiber-optic sensor for reference purposes. Other techniques to measure the temperature with a macro-bend sensor have been developed using either single mode [5] or multimode [7,18] silica optical fiber. The main advantages of using POF instead of glass fiber to build the sensor are the large core diameter and smaller Young modulus, making them less fragile and easier to handle; reducing development and maintenance costs. Although polymer based sensors have a smaller temperature range. The highly multimode nature of POF sensors implies especial features as the influence of external surrounding media that have to be considered in practical implementations.

In this context, the purpose of this work is to design and implement a POF [8] temperature sensor in a self-reference configuration and including a comprehensive experimental analysis to provide insight into the effect of different surrounding media and bend radii on the sensor response. Experimental results are also compared with mathematical models based on geometric approaches [18].

## 2. Principle of Operation

**Figure 1.**
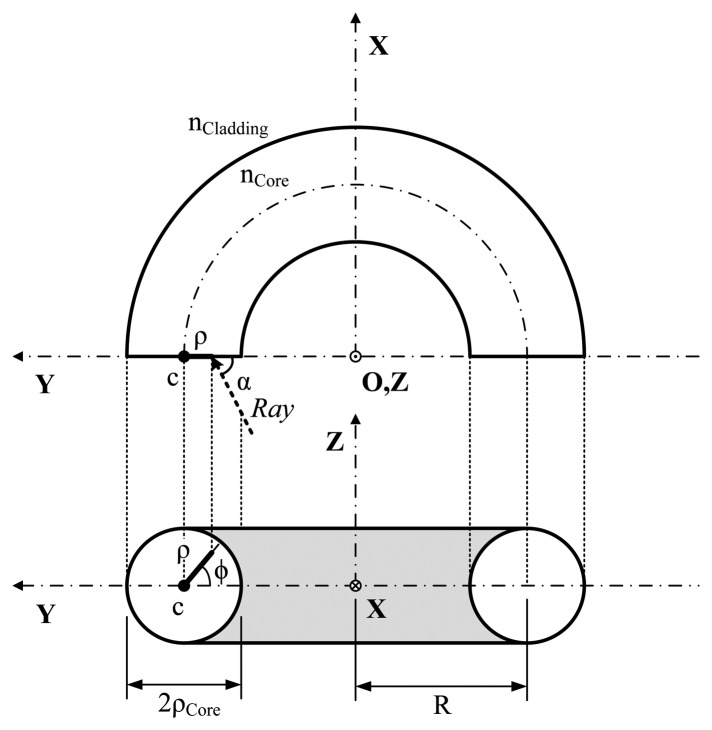
Schematic of a bend fiber section curved with a radius of curvature R.

Figure 1 shows a schematic of a curved multimode step index optical fiber [19]. The radius of curvature is R and the core diameter is 2ρ_Core_. The refractive indices of the core and cladding are n_Core_ and n_Cladding_, respectively. Optical power is launched at the beginning of the rectilinear region of the fiber. The geometrical description of the core rays in a step-index optical fiber is more complex than in a planar waveguide [20], due to the presence of the skew rays. The guidance of the core rays is achieved by ensuring that the propagation angle, α, satisfies the condition: 0 ≤ α ≤ α_c_, where the critical angle (α_c_) is given by: α_c_ = sin^−1^ (n_Cladding_/n_Core_) The expression of the numerical aperture (NA) for the rectilinear region is given by:

(1) $$NA=n_{\text{Core}}\cdot \sin\alpha \leq {\left(n_{\text{Core}}^{2}-n_{\text{Cladding}}^{2}\right)}^{1/2}$$

However, in the bend optical fiber, the guidance of the core rays can follow two ways. Only the rays entering the bent part of the fiber in the meridional plane remain with the same angle of incidence along a given ray path. On the other hand, the skew rays entering this plane, after the successive reflections within the core, do not follow a simple repeatable pattern because of the asymmetry introduced by bending the fiber. So when the optical fiber is bent, the local numerical aperture changes at a given location of the bent optical fiber. The dependence of the numerical aperture with the bend is given by [19]:

(2) $$NA(R,\rho ,\phi )=n_{\text{Core}}{\left[1-\frac{n_{\text{Cladding}}^{2}}{n_{\text{Core}}^{2}}{\left(\frac{R+\rho_{\text{Core}}}{R-\rho \cdot \cos\phi }\right)}^{2}\right]}^{\overset{1}{\;}/\underset{2}{\;}}$$

where ϕ is the ray angle at the beginning of the bend, which varies from 0° to 180°, ρ_Core_ is the fiber core radius and ρ is the radial position in the core satisfying the relation 0≤ ρ≤ ρ_Core_.

The optical fiber sensor proposed in this paper is based on a macro-bend POF. In this intensity sensor, the losses induced by the bending effect depend on the numerical aperture, that change with temperature. The refractive index of the core and cladding POF depend on the temperature. The POF used in the experiments is a Rayon^®^ Eska^®^ SH-4001 (Mitsubishi, Tokyo, Japan) with core and cladding manufactured using polymethyl methacrylate (PMMA) and fluorinated polymer, respectively. The temperature dependence of the core refractive index can be expressed as [21]:

(3) $$n_{\text{Core}}(T)=K_{2}\cdot T^{2}+K_{1}\cdot T+n_{0}$$

where K_1_= −1.15·10^−4^(°C)^−1^ is the thermo-optic (TO) coefficient of the core, K_2_= −5.173·10^−7^(°C)^−2^ is the second order temperature dependence term of the core and n_0_=1.49538 is the core refractive index at 0 °C. On the other hand, the temperature dependence of the cladding refractive index is given by [22]:

(4) $$n_{\text{Cladding}}(T)=n_{\text{Cladding}}(T_{0})+K_{3}\cdot (T-T_{0})$$

where K_3_= −3.5·10^−4^ (°C)^−1^ is the TO coefficient of the cladding and n_Cladding_(T_0_)=1.403 is the cladding refractive index at the reference temperature (T_0_ = +25 °C).

It can be seen that |K_3_| > |K_1_|. Finally, from Equation (2), the local numerical aperture in the bent section of the fiber *versus* the temperature can be expressed as:

(5) $$NA(T,R,\rho ,\phi )=n_{\text{Core}}(T){\left[1-\frac{n_{\text{Cladding}}^{2}(T)}{n_{\text{Core}}^{2}(T)}{\left(\frac{R+\rho_{\text{Core}}}{R-\rho \cdot \cos\phi }\right)}^{2}\right]}^{\overset{1}{\;}/\underset{2}{\;}}$$

**Figure 2.**
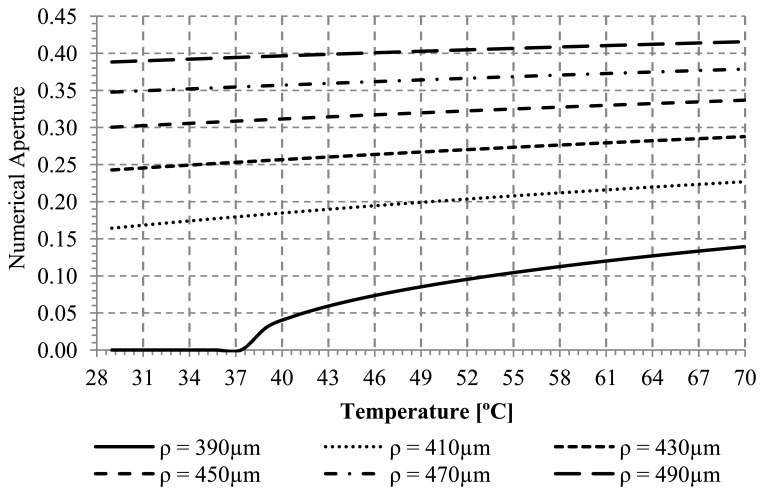
Local numerical aperture *versus* temperature for several radial positions in the core, with 2ρ_Core_ =980 μm, R=2.0 mm and ϕ=150°.

Using Equations (3) to (5), the temperature dependence of the numerical aperture for different ray radial positions in the core has been plotted in Figure 2.

**Figure 3.**
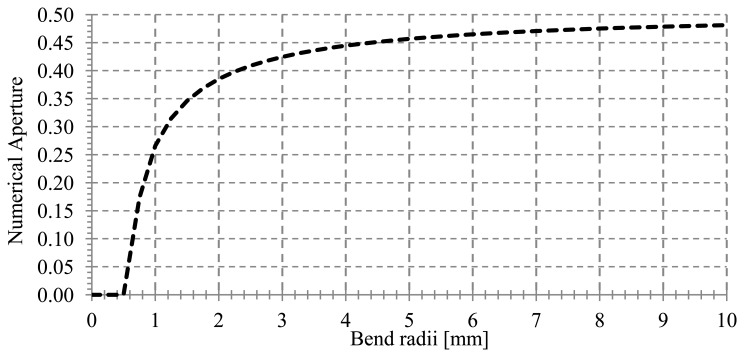
Local numerical aperture *versus* bend radius at *T* = +25°C, with 2ρ_Core_ = 980μm, ρ = 490 μm and ϕ = 150°.

Analyzing the positive slope of the curves for each radial position, it can be seen that the local numerical aperture increases when the applied temperature increases. This increment happens because an optical ray that is unguided at the reference temperature becomes guided at temperatures greater than the reference temperature. Figure 3 shows the local numerical aperture as a function of the bend radius at T = 25 °C. The local numerical aperture increases as the bend radius increases up to the local numerical aperture for a straight fiber (R = ∞) with a value around 0.5.

The insertion losses of the sensor at a specific temperature increase as the bend radius decreases. The POF used in the experiments has a critical radius of curvature of 25 mm, so the manufactured sensor is always operating at a lower R value as it is shown below.

## 3. Manufactured Sensor and Measurements

**Figure 4.**
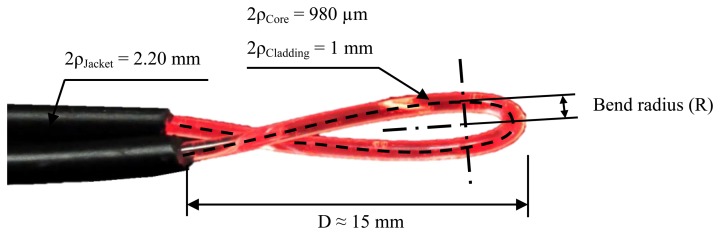
Schematic and photograph of the macro-bend POF sensor.

In this section, the sensor manufacturing process, the experimental set-up and the measurements of the sensor calibration curves are reported. In Figure 4, a photograph of the manufactured fiber-optic temperature sensor can be seen. A commercial step index POF fiber with a good tensile strength and flexing is used. These characteristics provide good mechanical properties at the time of manufacturing the sensor. From the middle section of the fiber length, the buffer coating is partially stripped. The length of this stripped section is about 30 mm. The fiber sensor is formed by creating a single 180° loop (½ turn) with a concrete bend radius, as shown in Figure 4. To fix the radius, a cylinder is used to facilitate and give more precision to the bending process. Then, the buffer coating in the junction of the two branches is fixed by cyanoacrylate adhesive. With this simple method, a stable macro-bend fiber temperature sensor can be manufactured.

**Figure 5.**
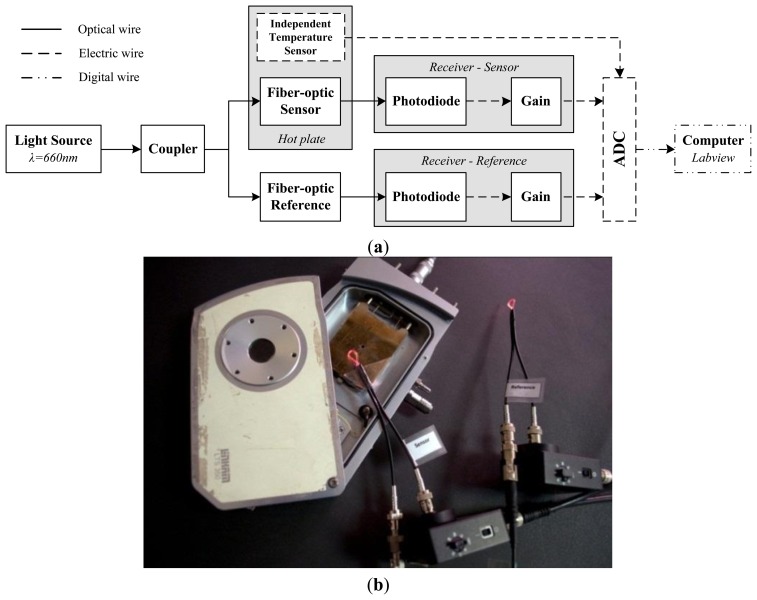
(**a**) Schematic of the experimental set-up for characterizing the POF temperature sensor. (**b**) Photograph of the experimental set-up with the sensing and referencing branch.

The schematic of the experimental set-up is shown in Figure 5a. The light source is a 660 nm Light-Emitting Diode (LED). The fiber-optic sensor was fixed close to the rectangular highly conductive metal base plate of a heating unit (LTS350, Linkam, Surrey, United Kingdom). The temperature of the hot plate was controlled with a controller unit (Linkam TP94). For measuring the real temperature of the optical fiber sensor, an independent electronic temperature sensor LM35 was used in the measurements. Temperature measurements are taken on the hot plate and at a 1 mm distance from the hot plate. A thermal isolated stage has been performed for supporting the fiber sensor when it is placed at a 1 mm distance from the plate. A caliper has been used to measure the position.

An additional macrobend fiber loop, identical to the proposed fiber-optic sensor is used for reference purposes, as shown in Figure 5. This fiber-optic reference or dummy sensor, placed outside of the heating unit, monitors the optical power fluctuations not related to the sensing magnitude. The output voltage at the reference (V_Reference_) and sensing (V_Sensor_) branch can be expressed as:

(6) VReference=βReference⋅ℜλ⋅δ⋅GReference⋅F'(T0)⋅PLight Source

(7) $$V_{\text{Sensor}}=\beta_{\text{Sensor}}\cdot {ℜ}_{\lambda }\cdot \delta \cdot G_{\text{Sensor}}\cdot F(T)\cdot P_{\text{Light Source}}$$

where β_Sensor_ and β_Reference_ are factors including the attenuation of the fiber leads, coupler insertion losses and connectors losses, of the sensing and reference branch respectively. 𝕽**_λ_** is the photodiode responsivity at the operating wavelength (λ = 660 nm), considering identical photodiodes. δ is the scale factor of the receivers for a specific output impedance. G_Reference_ and G_Sensor_ are the transimpedance amplifier gains for the reference and sensor branch, respectively. F(T) is the optical power modulation function *versus* temperature at the fiber-optic sensor and F'(T_0_) is the insertion losse at the reference temperature (T_0_) of the dummy sensor. Finally, P_Light Source_ is the optical power of the light source.

In order to compare the calibration curve with mathematical models and other intensity fiber optic sensors, the output voltage at the reference and sensing branch has been expressed in optical power units. The output optical power at the reference (P_Reference_) and sensing (P_Sensor_) branch can be expressed as:

(8) PReference=βReference⋅F'(T0)⋅PLight Source

(9) $$P_{\text{Sensor}}=\beta_{\text{Sensor}}\cdot F(T)\cdot P_{\text{Light Source}}$$

From both equations, the self-reference output power ratio γ_SR_ is defined as:

(10) γSR=PSensorPReference=βSensor⋅F(T)⋅PLight SourceβReference⋅F'(T0)⋅PLight Source=βSensorβReference⋅F'(T0)⋅F(T)

Therefore the ratio of the output optical powers depends on the power sensor variations with temperature and on a constant ratio between the insertion losses of the sensing and reference branch.

As previously stated, the conditioning circuit is made of a photodiode and a transimpedance amplifier, see Figure 5a. The amplifier gain was fixed at 10dB for all measurements.

In the experiments, the self-reference output power ratio (γ_SR_) has been measured from +29 to +70°C at 2 °C intervals. The minimum temperature has been limited by the cooling capabilities of the heating unit and the maximum temperature was limited by the capabilities of the POF. A time interval of 2 min has been set, between each temperature measurement, to ensure the stabilization of the voltage values. The sample rate and the number of samples of the output power ratio at each temperature have been fixed at 5 Hz and 10 samples, respectively. A total of six sets of measurements of the calibration curve have been carried out to perform a complete statistical analysis of the sensor parameters. Software based on National Instrument Labview^®^ code has been developed to acquire the voltage from the receivers, to calculate the optical power of each branch and to post-process the data.

### 3.1. Temperature Sensor Characterization for Different Radii

**Figure 6.**
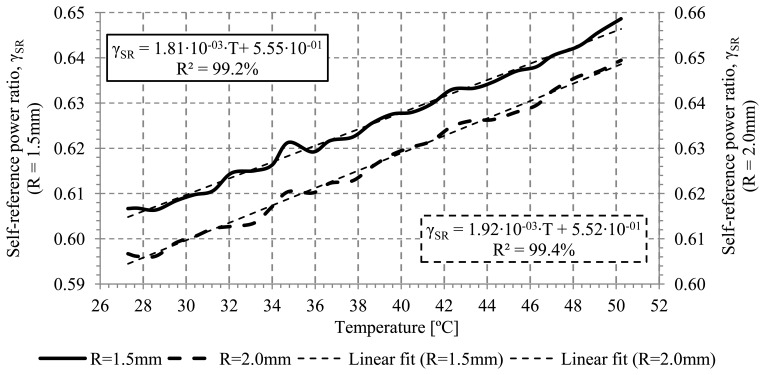
Self-reference output power ratio *versus* temperature for different bend radii.

Figure 6 shows the measured calibration curves for a bend radius of 1.5 and 2.0 mm. The distance between the sensor and the hot plate was fixed at 1.0 mm. Setting a distance between the sensor and the hot plate prevents possible changes in the refractive index of the cladding due to the contact with other materials as it is discussed in the next section. At a 1.0 mm distance, the temperature of the sensor varies from +27.2 to +50.2 °C.

The measurements show a good linearity response. The sensitivity obtained for the two bend radii was 1.81·10^−3^ and 1.92·10^−3^ (°C)^−1^ for a bend radii of 1.5 and 2.0 mm, respectively. These values can be expressed as 1.27·10^−2^ dB/°C and 1.29·10^−2^ dB/°C. From these values, it is observed that the sensor temperature sensitivity is clearly higher than the temperature coefficient of the fiber attenuation β_R_=1.5·10^−6^ dB/°C, for a 3 cm long POF sensor [23]. Linear regression coefficients greater than 99% are obtained.

The experimental results have been compared with simulations using a mathematical model based on geometric approaches. The model is based on treating light as individual rays that propagate in a zigzag path, taking into account the changes in ray directions and probable radiation losses that take place at the reflection points on the core-cladding interface. A detailed description of the model can be seen in [19]. The simulation shows the ratio between the input and output power at the sensor (η) for 50,000 rays and an infinite cladding interface as in [19].

In order to compare the measured calibration curves with the simulations, it has been derived the measured input and output optical power at the sensor curve.

**Figure 7.**
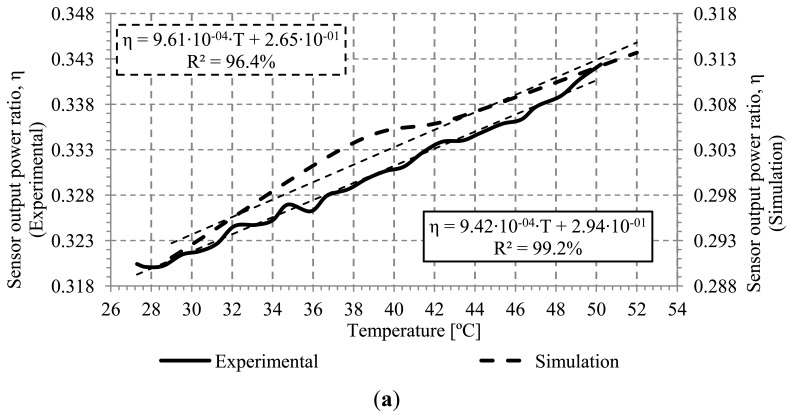

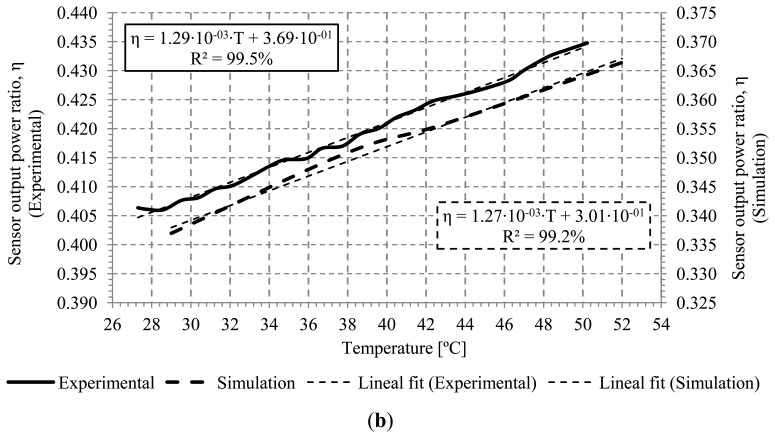
Simulations and experimental sensor output power ratio for several bend radius. (a) 1.5 mm; (b) 2.0 mm.

The simulations and measurements for bend radii of 1.5 and 2.0 mm can be seen on Figure 7. Four temperatures from 29 to 52 °C has been evaluated to get the theoretical calibration curve of the sensor in both cases.

Figure 7a shows the theoretical and experimental sensitivity for a bend radius of 1.5 mm. The values obtained were 9.61·10^−4^(°C)^−1^ and 9.42·10^−4^(°C)^−1^, respectively. The linear regression coefficients were 96.4% and 99.2%, respectively. On the other hand, Figure 7b shows the theoretical and experimental sensitivity for a bend radius of 2.0 mm, with values of 1.27·10^−3^(°C)^−1^ and 1.29·10^−3^(°C)^−1^ respectively. The linear regression coefficients are higher than 99%, in both cases. The insertion losses of the macro-bend sensor without connectors for bend radii of 1.5 and 2.0 mm are 4.94 and 3.91 dB, respectively. As expected, the insertion losses for a bend radius of 1.5 mm are greater than those for a bend radius of 2.0 mm. It can be concluded that measurements are in good agreement with simulations, with better results for a 2 mm bend radius. Analyzing the sensitivity for the proposed bend radii, it can be seen that it increases when bend radius increases, due to the increment of the numerical aperture with bend radius, as previously shown in Figure 3

### 3.2. Temperature Sensor Characterization on Different Surrounding Media

The step index POF fiber is highly multimode, so part of the light propagates through the cladding, but the cladding is only around 20 μm thin, so the external medium with a specific refractive index can influence the propagation through the fiber. The practical implementation of the sensor implies different manufacturing procedures and testing, and different surrounding media related to them.

**Figure 8.**
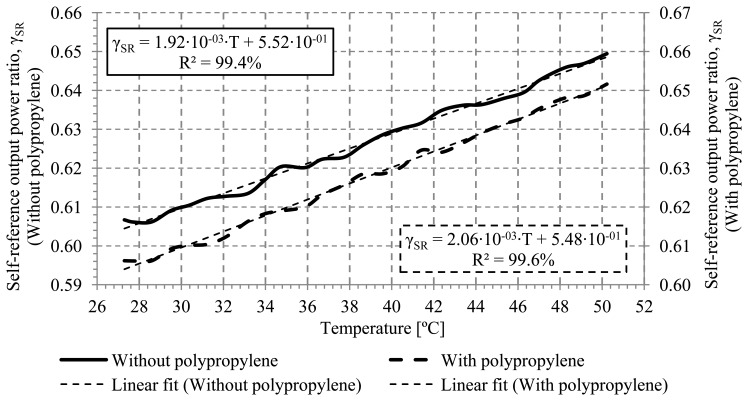
Calibration curves for a sensor with and without tape coating.

One possible disturbance of the refractive index is the use of adhesives on the probe to fix the temperature sensor on the sensing point. These adhesives typically are manufactured with polymeric materials and have a different refractive index to that of the fiber cladding. In these experiments, the adhesive used to cover the fiber cladding of the sensing area was a polypropylene tape whose refractive index (n_Polypropylene_ = 1.515) [24] is higher than the refractive index of the cladding. Figure 8 shows the calibration curves for a sensor with and without the tape. In these measurements, the distance between the optical sensor and the hot plate was fixed at 1.0 mm to avoid any possible contact with the copper plate. The bend radius of the optical sensor was fixed at 2.0 mm. The sensitivity obtained with and without the tape was 2.06·10^−3^(°C)^−1^ and 1.92·10^−3^(°C)^−1^, respectively. In both cases, the linear regression coefficient is greater than 99%. It can be inferred that with and without the tape the results can be considered the same within the error.

**Figure 9.**
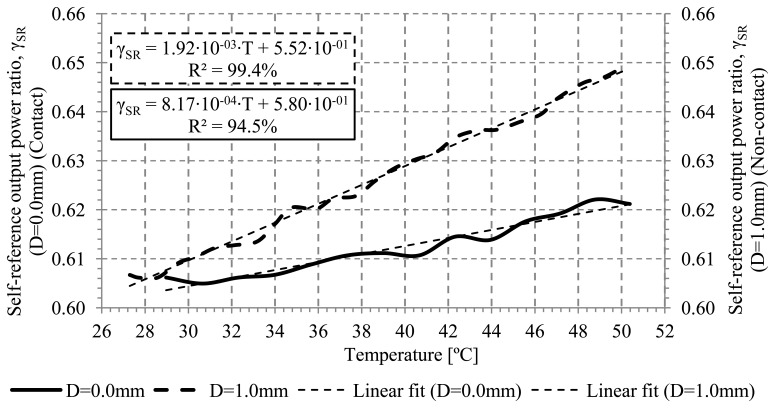
Calibration curves for a sensor with no contact and in contact with the metallic surface of the hot plate.

Another important parameter to consider is the possible influence of the hot plate metal surface. Placing the sensor on the surface can cause changes of the refractive index surrounding the cladding. In our experiments, the heater for measuring the calibration curves is a hot plate made of copper. The refractive index of the copper (n_Copper_=0.23) [25] is much lower than the refractive index of the cladding. To analyze its influence, it has been measured the output voltage ratio in two scenarios: non-contact and in contact with the hot plate. The distance to ensure non-contact between the sensor and the hot plate has been fixed at 1.0 mm. On other hand, for this experiment, no tape has been used in order to isolate the phenomenon to be analyzed. The bend radius of the optical sensor is 2.0 mm. Measurements are shown on Figure 9, it can be seen that the sensitivity at both scenarios was 1.92·10^−3^(°C)^−1^ and 8.17·10^−4^(°C)^−1^, for in contact and at a 1.0 mm distance respectively. The sensitivity is reduced by a factor of around 2.4 when the POF is in contact with copper, a metal with an imaginary refractive index. The linear regression coefficients for the two measured distances are 99.4% and 94.5%, respectively. The contact with the metal surface is the responsible of the additional loss, having in mind that the cladding thickness is sufficiently small that the evanescent field of the core modes may extend through the cladding into the jacket. If the cladding is lossless, all the attenuation takes place in the lossy metal jacket. The power distribution ratio of the cladding to the total power changes with the cladding thickness and the temperature [26].

## 4. Discussion

In this article, the authors have shown a temperature sensor based on a POF macro-bend loop. Different experiments have been carried out for measuring the calibration curves at different conditions. To ensure the stability of the sensor over time, six sets of measurements were carried out with a time interval between them of about 2 h. For a bend radius of 2.0 mm, the temperature sensor system has a calibration curve whose sensitivity is 1.92·10^−3^(°C)^−1^. A statistical analysis of the calibration curve shows that the sensor has a linear response with a non-linearity full scale error of 3.9%. The designed system allows a resolution of 0.3 °C from +27.2 to +50.2 °C.

The most limiting component in the system resolution is the data acquisition card. The acquisition card in this experiment is a USB 6009 (National Instruments^®^, Austin, TX, United States of America) with a noise value of 0.5 mV_RMS_ at a voltage range of ±1.0 V. Using another acquisition card, the resolution of the system can be reduced to obtain a value of 0.1 °C being limited by the noise generated by the transimpedance amplifier gain.

The optical input and output power measured at the sensor curve are compared with the simulations. Those values are obtained from measuring the optical power at the input and output of the sensor and estimating the connector, adaptor and straight section fiber losses. In the measurements, two types of connectors have been used from different manufacturers and with different losses. For a bend radius of 1.5 mm and 2.0 mm, the connector losses are measured to be 1.2 and 2.2 dB, respectively. These values are below the maximum limits (≤2.5 dB) set by the two manufacturers. The self-reference topology proposed avoid the sensor response dependence of these connector losses and of other losses that can be present under the sensor operation.

The intensity sensors are usually used at non-critical and low-cost systems when high precision and resolution are not required. A comparative analysis has been developed between the proposed sensor and other intensity temperature sensors reported on the literature (see Table 1).

From Table 1, it can be seen that the different sensor parameters are similar, apart from the sensitivity. The sensitivity of the proposed sensor is similar to that obtained with silica fibers [5,7,13], but they are also more fragile and usually needs more expensive equipment. The other sensors based on fluorescence techniques, measure the attenuation of the luminescence signal caused by the presence of a convenient indicator, such as glue or ink [13,14]; they can obtain a higher sensitivity but making them also more complex approaches.

In terms of reproducibility, as in any intensity sensor it is essential to have a self-reference configuration to avoid the influence of external loss variations of the whole set-up. Special care should be taken with connector losses, and with the manufacturing process of the fiber-optic sensor.

On the other hand, the experimental results show the effect of the external environment on the temperature measurement. As described previously, changes in the refractive index of the surrounding media in contact with the cladding, especially metals, produce a reduction in a factor of more than two in the sensor sensitivity. For the proper operation of the proposed temperature sensor, it is very important to carry out a characterization of the sensor under real working conditions. This property can also be used to improve the system but at the expense of increasing its complexity. As shown in Table 1, different sensors use absorbed coatings that modify the refractive index of the fiber to increase the sensitivity of the sensor and thereby improving the sensor parameters. Finally, this behavior can also be used to develop other type of sensors, as for example a detector of different metal materials in industrial processes.

## 5. Conclusions

In conclusion, macrobending loss, caused by the different thermo-optic coefficients of the cladding and core of a step-index Polymer Optical Fibers (POFs) has shown to be a usable technique for temperature sensing. The sensor has been tested in the temperature range from +27.2 to +50.2 °C. The sensor sensitivity for a bend radius of 2.0 mm is 1.29·10^−3^(°C^−1^). The sensitivity increases as the radius of curvature increases. In addition, sensor sensitivity measurements are in good agreement with simulations. The automated self-reference sensor system shows a resolution below 0.3 °C and a 3.9% non-linearity full scale error. The proposed sensor requires only a few and relative low-cost components. The influence of external media in contact with the sensor has been analyzed. The sensor can be used in a large range of applications, for instance: instrumentation process, automotive industry, air-conditioning control, chemical industry, biomedical sector and detection of metal particles in contaminated fluids.

## Figures and Tables

**Table 1.** Comparative table with other intensity fiber-optic temperature sensors.

	
	**Fiber**	**Range (Note 1)**	**Sensitivity (Note 2)**	**Resolution**	**Linear Regression Coefficient**

Macro-bend loss [5]	Singlemode Silica fiber	0 to +75 °C	R = 12.5 mm: 1.2·10^−02^ dB/°C	<1 °C	(Note 3)

Modified macro-bend loss [7]	Multimode Silica fiber	Olives oil: +55 to +85 °C Gasoline: +25 to +55 °C	Olive oil: 1.5·10^−02^ dB/°C Gasoline: 2.3·10^−02^ dB/°C	0.1 °C	(Note 3)

Fluorescence and glue [13]	Multimode Silica fiber	+25 to +100 °C	≈1.0·10^−02^ dB/°C	2 °C	>99%

Fluorescence and glue [14]	Multimode POF	+22 to +73 °C	≈8.4·10^−02^ dB/°C	(Note 3)	>99%

Macro-bend (Proposed)	Multimode POF	+27.2 to +50.2 °C	R = 2.0mm: 1.29·10^−02^ dB/°C (Note 4)	0.3 °C	>99%

Notes: 1. Range on the reported measurements. For example, in our prototype the real temperature range for this sensor is from +29 to +70 °C; 2. Approximate values obtained from the graphs of the articles; 3. This value is not shown in the article; 4. Self-reference system sensitivity.
